# Multiple-locus variable-number tandem repeat analysis for genotyping of erythromycin-resistant group B streptococci in Iran

**DOI:** 10.1016/j.nmni.2022.100957

**Published:** 2022-01-17

**Authors:** Mahsa Ghamari, Fereshteh Jabalameli, Mohammad Emaneini, Reza Beigverdi

**Affiliations:** 1) Department of Microbiology, School of Medicine, Tehran University of Medical Sciences, Tehran, Iran; 2) Medical Mycology and Bacteriology Research Center, Kerman University of Medical Sciences, Kerman, Iran

**Keywords:** *ermB*, *ermTR*, Erythromycin, GBS, MLVA

## Abstract

**Background:**

Group B Streptococcus (GBS or *S. agalactiae*) is an important pathogen causing severe invasive diseases in neonates, pregnant women, and adults with underlying medical conditions.

**Methods:**

To investigate the incidence of resistance to macrolide, lincosamide and streptogramin type B (MLS_B)_ antibiotics, macrolide and tetracycline resistance determinants and genetic relationships, a total of 146 clinical isolates of GBS were collected from Tehran, Iran. The genetic relationships between erythromycin-resistant strains were determined by multilocus variable tandem repeat analysis (MLVA).

**Results:**

All isolates were susceptible to penicillin, vancomycin, linezolid, and quinupristin–dalfopristin, but were resistant to tetracycline (96.6%, 141/146), erythromycin (28.1%, 41/146) and clindamycin (16.4%, 24/146). Among the 41 erythromycin-resistant GBS (ERGBS), the most common antimicrobial resistance gene was *tetM* detected in 92.7% (38/41) of the isolates followed by *ermTR* and *ermB* found in 65.8% (27/41) and 29.3% (12/41) of isolates, respectively. Of the 41 ERGBS, 95% (39/41) exhibited the constitutive MLS_B_ phenotype, 2.4% (1/41) displayed inducible MLS_B_ and 2.4% (1/41) had M phenotype. The *erm* methylase genes were widely related to MLS_B_ phenotype isolates, while the *mefA* gene was associated with M phenotype. MLVA analysis performed on the 41 ERGBS revealed that 34 MLVA types (MTs). MLVA analysis showed that infections due to ERGBS have been caused by a variety of genotypes, suggesting that ERGBS were clonally unrelated and dissemination of these isolates was not due to a clonal outbreak.

**Conclusion:**

Careful usage of macrolide antibiotics in therapy, continued surveillance of resistance rate and appropriate infection control measures can help to reduce spreading of resistance isolates.

## Introduction

Group B Streptococcus (GBS or *S. agalactiae*) is an important pathogen that may cause severe infection in the neonates, pregnant women, elderly, and immunocompromised individuals [1]. In neonates and infants, GBS infections are separated into early-onset disease (EOD; birth to 6 days) and late-onset disease (LOD; 7 to 89 days) [2]. The clinical manifestations of GBS infection vary greatly and include sepsis, pneumonia, meningitis, endometritis, skin or soft tissue and infections, urinary tract infection, endocarditis and arthritis [1,2]. The case fatality rate for GBS infection in elderly adults is approximately 15%, remarkably higher than the 4%–6% reported for neonates with invasive GBS disease [3]. Penicillin has been established as a first line antibiotic for the treatment of GBS infections [4]. However, macrolide, lincosamide and group B streptogramins (MLS_B_) antibiotics have been recommended as appropriate alternative agents for patients who are allergic to beta lactam agents [5]. Macrolide resistance in GBS is due either to ribosomal methylation or efflux pumps [5,6]. Ribosomal modification encoded by *erm* genes (*ermA/TR* and *ermB*) is associated with co-resistance to MLS_B_ antibiotics with high-level resistance to all MLS_B_ antibiotics [5,6]. Phenotypic expression of MLS_B_ resistance can be constitutive (cMLS_B_) or inducible (iMLS_B_) [5,6]. Efflux-mediated resistance encoded by *mef* genes is related to the M phenotype and resistance only to 14- and 15-member ring macrolides [5,6]. In order to understanding genetic relationships and population structure of GBS, several molecular typing methods have been developed among which multiple locus variable number tandem repeat analysis (MLVA) has high discriminatory power for differentiating between related and unrelated strains [7]. The aim of this study was to determine the prevalence of macrolide resistance in GBS and to investigate their resistance phenotypes and clonal relationships.

## Materials and methods

### Bacterial isolates

Between July 2013 and February 2014, in a cross-sectional study, a total of 146 nonduplicated GBS isolates were collected from three hospitals (Imam Khomeini hospital, Baqiyatallah hospital and Pars hospital) in Tehran, Iran. Patients ranged in all age groups; 13 and 6 isolates were recovered from throat and ear of newborns of pregnant women at gestational age 35–37 weeks and these newborns did not develop EOD or LOD. One hundred twenty-seven strains were isolated from pregnant and non-pregnant patients. Isolates were collected from different sources. Majority of them were from urine (*n* = 121), wounds (*n* = 3), and fluids (*n* = 3). Each isolate belonged to a separate patient. All isolates were re-identified using standard microbiological techniques including gram stain, catalase, CAMP and hippurate hydrolysis Tests [8]. To confirm the identity of isolate as GBS, the *dltS* gene was targeted by polymerase chain reaction (PCR) [9].

### Antimicrobial susceptibility testing

The disk diffusion method was performed for clindamycin (2μg), erythromycin (15μg), vancomycin (30μg), linezolid (30μg), penicillin (10 unites), tetracycline (30μg), and quinupristin-dalfopristin (15μg) according to the Clinical and Laboratory Standards Institute (CLSI) guidelines [10]. *Enterococcus faecalis* ATCC 29212 was used as a control strain. The constitutive, inducible and M resistance phenotypes were determined by a double-disk test with erythromycin and clindamycin, as described previously [11].

### DNA extraction

The genomic DNA was extracted from all isolates using the Gene All Exgene™ Cell SV (Gene ALL, Seoul, Korea), according to the manufacturer's instructions.

### Detection of antimicrobial resistance genes

The genes encoding resistance to the MLS_B_ antibiotics (*ermA*, *ermB*, *ermC*, *ermTR*, *mefA* and *linB*) and tetracyclines (*tetM*, *tetL*, *tetK* and *tetO*) were investigated by PCR as described previously [[12], [13], [14]].

### MLVA typing

MLVA analysis of GBS was performed only on erythromycin resistant strains as described previously by PCR amplification of five loci (SATR1-SATR5) containing tandem repeats [7]. The PCR program for all loci was performed under the following conditions: initial activation at 95°C for 5 min, followed by 30 cycles at 94°C for 30 s, 60°C for 90 s, and 72°C for 60 s, and a final extension at 72°C for 10 min. The PCR products were electrophoresed in a 1.5% agarose gels with 0.5X TBE (Tris/Borate/EDTA) buffer. The DNA bands were visualized by KBC power load dye staining and photographed under UV illumination. The number of repeats in each locus was calculated by subtracting the sizes of the flanking regions from the amplicon size and then dividing by the size of the repeat unit [7]. The result was rounded down to the nearest complete copy number. An unweighted pair group method with arithmetic mean (UPGMA) dendrogram based on MLVA profiles of GBS was created by PHYLOViZ 2.0 software [15]. GBS isolates that differed in one or more than one of the five loci were considered distinct MLVA types (MTs) [16].

## Results

**Fig. 1:**
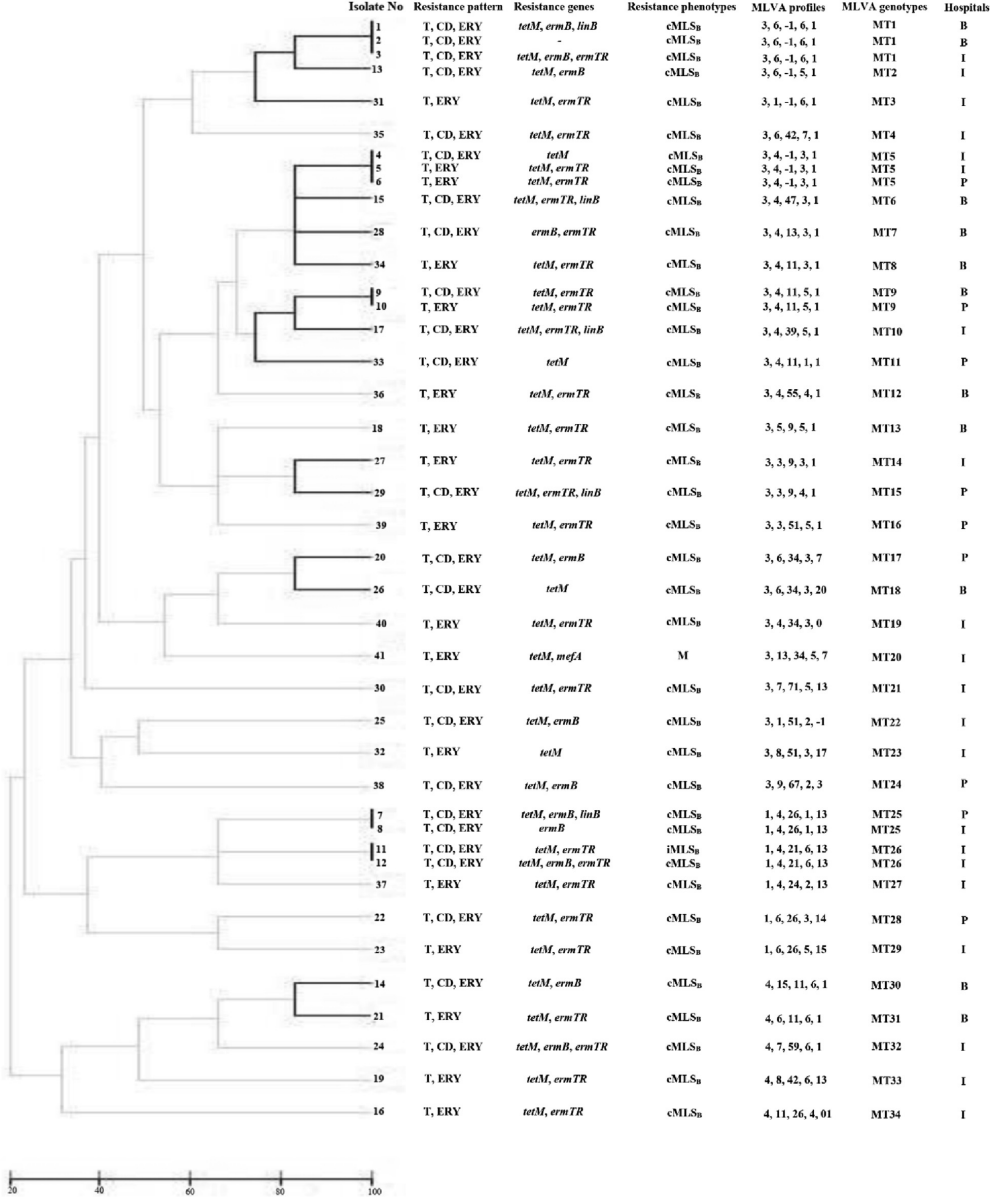
The phenotypic and genotypic characteristics of 41 erythromycin-resistant GBS isolates included in the present study. Each MLVA type (MT) (*n* = 34) is presented. cMLSB: Constitutive macrolide–lincosamide–streptograminB resistance phenotype, iMLSB: Inducible MLSB, M- Phenotype: Macrolide resistance phenotype. I: Imam Khomeini hospital, B: Baqiyatallah hospital, P: Pars hospital.

All isolates were susceptible to the penicillin, vancomycin, linezolid and quinupristin-dalfopristin. Resistance to tetracycline, erythromycin and clindamycin was detected as 96.6% (141/146), 28.1% (41/146) and 16.4% (24/146) of strains, respectively. Among the 41 ERGBS, 95.1% (39/41), 2.4% (1/41) and 2.4% (1/41) strains showed the cMLS_B_, iMLS_B_ and M phenotypes, respectively (Fig 1). All the ERGBS were concurrently resistant to tetracycline. The most prevalent gene was *tetM* found in 92.7% (38/41) of the isolates followed by *ermTR*, *ermB*, *linB*, and *mefA* 65.8% (27/41), 29.3% (12/41), 12.2% (5/41), and 2.4% (1/41) of isolates, respectively. The *ermTR* and *ermB* genes were detected in 48.8% (20/41) and 14.6% (6/41) isolates with the cMLS_B_ phenotype, respectively. The *ermTR*/*ermB*, *ermTR*/*linB* and *ermB*/*linB* genotypes were present in 9.7% (4/41), 7.3% (3/41) and 4.9% (2/41) isolates with the cMLS_B_ phenotype, respectively. One iMLS_B_ phenotype and one M phenotype had *ermTR* and *mefA*, respectively (Fig 1). Five and three isolates did not carry any of tested the macrolide and tetracycline resistance genes, respectively. The *ermA*, *ermC*, *tetL*, *tetK* and *tetO* genes were not found in any isolates. All ERGBS (n = 41) were subjected to strain typing by MLVA. The results of MLVA analyses are shown in Fig. 1. This method revealed that our isolates were genetically diverse and highly heterogeneous. According to the dendrogram (Fig. 1), MLVA analyses displayed 34 MTs or different allelic profiles. Five MTs were displayed by more than 1 isolates: MT1 (n = 3), MT5 (n = 3), MT9 (n = 2), MT25 (n = 2) and MT26 (n = 2). Twenty-nine MTs were presented by only 1 isolate (Fig 1).

## Discussion

Our data revealed that all isolates were susceptible to penicillin, vancomycin, linezolid and quinupristin-dalfopristin. These results are in agreement with reports from other authors [17,18] and confirm that the use of penicillin is still recommended as the first therapeutic agent for treatment of GBS infections. However, reduced penicillin susceptibility strains have been documented in Japan, Hong Kong and USA [[19], [20], [21]]. A high rate of tetracycline resistance (97.6%) was observed in our study. This finding is in line with other studies performed in different countries such as Tunisia (97.3%) and the USA (96%) [22,23], but more than France (88.1%), Italy (80%), and Kuwait (89.5 %) [18,24,25]. Although tetracycline has not been used for the therapy of GBS infections, selective pressure due to intensive use of tetracycline to treatment of a wide variety of human and animal infections may have led to the emergence this resistance among GBS isolates [11]. In agreement with other studies, we observed that *tetM* accounts for the majority (92.7%) of tetracycline resistance [23,25,26]. The rate of erythromycin resistance in our study was 28.1%, which is higher than those reported from Germany (12%), Belgium (16.7%), Spain (8–18%), Italy (19.5%) and France (18–21.4%) [18,[27], [28], [29], [30]], but it was lower than the rate reported from USA (54%) and Taiwan (46%) [[31], [32], [33]]. Clindamycin resistance rate (16.4%) was in agreement with the resistance rates reported from New Zealand (15%) [32]. The widespread usage of macrolide is a major contributing factor leading to antibiotic resistance in our hospital settings [34]. Moreover, none of these hospitals had an active antibiotic stewardship guidelines and infection control measures. Unfortunately, in most Iranian hospitals, infection control team may exist on paper, in practice, they barely exist. According to the Centers for Disease Control and Prevention guidelines, all pregnant women should be screened for GBS with vaginal and rectal cultures between 35 and 37 weeks' gestation and should receive intrapartum antibiotic prophylaxis (IAP) with penicillin or ampicillin for culture-positive women [35]. Unfortunately, maternal screening for GBS in the 35–37th week of gestation has not implemented in Iran and no accurate estimate of the true burden of GBS disease was available in our country [36]. Seale et al. in their global systematic review reported that GBS is responsible for 205,000 cases of EOD, 114,000 cases of LOD and 33,000 cases of invasive disease in pregnant and postnatal women [37].

In our study, the vast majority of the ERGBS isolates exhibited the cMLS_B_ phenotype (95%, 39/41) and harbored *ermTR,* either alone or in association with *ermB* or *linB* genes. Similar finding was observed in Canada, where the *ermTR* and *ermB* genes were the major resistance mechanism [11]. In contrast, the M phenotype encoded by *mefA* gene was more frequently found in Taiwan [38] and iMLS_B_ phenotype encoded by *ermTR* gene was more commonly reported in USA [14]. This discrepancy may be related to the different patterns in use of antimicrobials, which led to the variation of resistant phenotypes [39]. The low prevalence of the *mefA* gene (2.4%) in our study is similar to that reported in the Tunisia, where 2.2 % of erythromycin-resistant GBS strains harbored this gene [23]. Similar to previous reports, combinations of macrolide and tetracycline resistance genes were observed in the current study [23,26]. Acquisition of resistance genes to erythromycin and tetracycline in GBS is generally associated with the presence of mobile genetic elements such as plasmids and conjugative transposons [40,41]. Molecular typing is a powerful tool in epidemiologic studies for determining the identical or closely related strains and sources of infection [42]. MLVA typing results showed a high level genetic diversity among our isolates. In our study, MLVA differentiated 41 ERGBS strains into 34 genotypes, suggesting that ERGBS were clonally unrelated and dissemination of ERGBS isolates was not due to a clonal outbreak. In our previous study, the MLVA scheme differentiated the 41 strains isolated from pregnant women into 30 genotypes [43]. Different MTs have been reported from studies in other countries [7,44,45]. Otaguiri *et al.* classified 83 Brazilian GBS strains into 15 genotypes [45]. Haguenoer *et al.* classified 186 French GBS strains into 98 genotypes [44]. Typically, isolation of many resistant bacteria in hospitals can be driven by two epidemiological patterns: the emergence and spread of a particular clone, or the persistence and co-existence of polyclonal lineages [46]. Our data are in agreement with the latter scenario, because many different MTs were observed in three hospitals and isolates from the same MTs were identified in different hospitals (Fig. 1). Similar finding was observed in Taiwan, where multiclonal spread was responsible for resistance to erythromycin in GBS population [39].

It should be emphasized that this study has several limitations, including lack of risk factors, demographics and clinical features of the patients, the relatively small number of ERGBS isolates compared to other studies with large scale studies and the lack of other molecular typing data such as pulsed-field gel electrophoresis or multi-locus sequence typing for further genotypic characterization of the these isolates.

In conclusion, our results show that erythromycin resistance is relatively high and the most common phenotype among GBS isolates was cMLS_B_ phenotype mediated mainly by the *ermTR* and *ermB* genes, respectively. MLVA analysis showed that infections due to ERGBS have been caused by a variety of genotypes. Thereby, the implementation of strict infection control, careful usage of macrolide antibiotics in therapy and continued surveillance of resistance rate should be continued in Iran.

## Authors’ contributions

RB and FJ designed the experiments. MG conducted the experiments, RB drafted the manuscript. ME revised the manuscript. All authors read and approved the final manuscript.

## Conflict of interest

The authors have no conflicts of interest to declare.
